# Utilizing genomic signatures to gain insights into the dynamics of SARS-CoV-2 through Machine and Deep Learning techniques

**DOI:** 10.1186/s12859-024-05648-2

**Published:** 2024-03-27

**Authors:** Ahmed M. A. Elsherbini, Amr Hassan Elkholy, Youssef M. Fadel, Gleb Goussarov, Ahmed Mohamed Elshal, Mohamed El-Hadidi, Mohamed Mysara

**Affiliations:** 1 Bioinformatics Group, Center for Informatics Science, School of Information Technology and Computer Science, Nile University, Giza, Egypt; 2 Microbiology Unit, Belgian Nuclear Research Centre (SCK•CEN), Mol, Belgium

**Keywords:** SARS-CoV-2, Genomic signature, Di nucleotide frequency, Tri nucleotide frequency, GenoSig, Deep Learning, Machine Learning, Random Forest

## Abstract

The global spread of the SARS-CoV-2 pandemic, originating in Wuhan, China, has had profound consequences on both health and the economy. Traditional alignment-based phylogenetic tree methods for tracking epidemic dynamics demand substantial computational power due to the growing number of sequenced strains. Consequently, there is a pressing need for an alignment-free approach to characterize these strains and monitor the dynamics of various variants. In this work, we introduce a swift and straightforward tool named GenoSig, implemented in C++. The tool exploits the Di and Tri nucleotide frequency signatures to delineate the taxonomic lineages of SARS-CoV-2 by employing diverse machine learning (ML) and deep learning (DL) models. Our approach achieved a tenfold cross-validation accuracy of 87.88% (± 0.013) for DL and 86.37% (± 0.0009) for Random Forest (RF) model, surpassing the performance of other ML models. Validation using an additional unexposed dataset yielded comparable results. Despite variations in architectures between DL and RF, it was observed that later clades, specifically GRA, GRY, and GK, exhibited superior performance compared to earlier clades G and GH. As for the continental origin of the virus, both DL and RF models exhibited lower performance than in predicting clades. However, both models demonstrated relatively higher accuracy for Europe, North America, and South America compared to other continents, with DL outperforming RF. Both models consistently demonstrated a preference for cytosine and guanine over adenine and thymine in both clade and continental analyses, in both Di and Tri nucleotide frequencies signatures. Our findings suggest that GenoSig provides a straightforward approach to address taxonomic, epidemiological, and biological inquiries, utilizing a reductive method applicable not only to SARS-CoV-2 but also to similar research questions in an alignment-free context.

**Supplementary Information:**

The online version contains supplementary material available at 10.1186/s12859-024-05648-2.

## Introduction

Unequivocally, the emergence of the severe acute respiratory syndrome coronavirus 2 (SARS-CoV-2) pandemic was the focus of the last three years. Over 6 million individuals have deceased on account of this pandemic by the time this article was written [1]. Briefly, SARS-CoV-2 is a beta coronavirus and the seventh member of the human coronaviruses (CoVs) [2, 3]. Four human CoVs (HCoV-229E, HCoV-NL63, HCoVOC43, and HCoV-HKU1) are able to cause mild, self-limiting upper respiratory infections, whereas SARS-CoV, MERS-CoV, and SARS-CoV-2 caused severe emergent outbreaks in 2002, 2012, and 2019 respectively [4]. Regarding the geographic distribution, as of the time of writing this research paper and based on data from the Coronavirus Observer project (https://covid.observer), Europe had the highest incidence of COVID-19 cases relative to its population, exceeding 220 million cases (~ 37% of its population). Asia exhibited more than 230 million cases (~ 4.9%), whereas North America documented over 120 million cases (~ 21%). South America's tally surpassed 67 million cases (~ 16%), Oceania recorded over 13 million cases (~ 33%), and Africa noted more than 12 million cases (~ 1%). It's important to highlight that even though North America ranks third in terms of overall COVID-19 case count, the United States bore the brunt of the pandemic's impact. The SARS-CoV-2 genome is organized into 16 nonstructural, 4 structural, and 9 accessory proteins [5]. Owing to its rapid replication, polymerase mistakes, host immune factors, and spontaneous damage, RNA viruses show a high rate of mutations, leading to high genetic variations and positive/negative selections of certain variants depending on the benefit of the variant for the viral evolution [6, 7].

Currently, according to GISAID clade stratification, SARS-CoV-2 genomes are classified by differing variants into 11 clades namely L, S, V, G, GH, GV, GR, GRY, GK, GRA, and genomes without any clear classification named as “O clade” [8]. These clades originated on different timeframes throughout the various distinct epidemic waves from the early split of S and L into V and G, which was followed by the division of G into GH (Beta), GR, and GV. More recently, GR has evolved into GRY (Alpha) and GRA (Omicron). Alongside temporal and phylogenetic diversity, clades are distinguished from each other by specific mutations, particularly those associated with certain structural variants, notably in the spike protein, such as D614G. Moreover, these clades display variations in the severity of infection. For instance, clades GR and GH were found to be more prevalent among individuals who experienced clinical deterioration, whereas clade GRA was associated with immune and vaccine escape, however, with less virulence [9–12].

Several efforts were made to investigate SARS-CoV-2 genomic data, including the utilization of machine learning (ML) for taxonomic classification and continental origins prediction [13, 14]. Regarding the taxonomic classification, Desai et al*.* introduced the Infectious Pathogen Detector (IPD), a web tool to perform genomic analysis and predict the phylogenetic tree clade from whole genome sequences raw data [15, 16]. Another tool introduced by Kaden et al*.* implements an alignment-free approach for RNA genomic analysis and combines it with a support vector classifier for virus evolution discriminated by amino acid changes [17]. Sawmya et al. also developed a model to predict the virulence of SARS-CoV-2 infection by classifying the genome sequences as either severe or mild [18]. Lastly, Lopez-Rincon et al*.* designed an automated pipeline to detect the SARS-CoV-2 deleterious variants in genome sequences [19]. Regarding the continental origin, two studies developed an ML framework for classifying SARS-CoV-2 sequences into their continental origins. Dlamini et al*.* managed to train classification models to distinguish between sequences of eight pathogenic species, including SARS-CoV-2, and distinguish between SARS-CoV-2 sequences originating from six continental regions by analyzing dinucleotide genomic signatures for whole genome sequence data [20]. Ekpenyong et al*.*—on the other hand proposed a computational approach for the identification of the continental origin of SARS-CoV-2 sub-strains and gender-specific isolates [21].

In this context, one important aspect of clade evolution is that it is a consequence of the synonymous and non-synonymous variants of the virus. While non-synonymous variants are more biologically crucial for protein evolution, synonymous variants can play a vital role in adaptation to the host [22]. In a phenomenon referred to as codon usage bias, viruses often display a preference for one of the synonymous codons, which leads to better adaptation to the host transcription system [23, 24]. Both synonymous and non-synonymous mutations significantly affect the genomic composition and subsequently what is known as the genomic signature. The latter notion was introduced initially by Karlin et al., in which prokaryotic species were characterized via the frequency of short oligonucleotides in their genomes, giving phylogenetic meaning patterns [25–27]. Recently, employing the notion of Karlin signatures, a tool named PaSiT was introduced as a fast straightforward method for measuring distances between related bacterial strains [28], providing large-scale comparison in a computationally friendly manner. Distinctions in Di nucleotide (Di) and Tri nucleotide (Tri) genomic profiles can be regarded as a unique genomic signature for particular taxonomic groups, offering valuable insights into the mechanisms of molecular evolution [29, 30].

Our objective was to extend the scope of the previous work on genomic signatures, such as that conducted by Dlamini et al*.* which had limitations in terms of dataset size (32,899 sequences) and the absence of an accessible standardized open-source tool for similar research inquiries. While their study focused solely on Di nucleotide frequency, our study aimed to establish a comprehensive framework for classifying distinct SARS-CoV-2 clades and inferring their geographic origins using Di nucleotide frequency combined with Tri nucleotide frequency. To achieve this, we re-implemented the PaSiT tool in a dedicated tool called GenoSig, which is more suitable for computing genomic signatures for genomes. We believe that this tool has the potential to become a standard method for generating Di and Tri nucleotide frequencies to train and test different ML and DL models.

## Material and methods

### Data collection

SARS-CoV-2 whole genome sequences (WGS) were downloaded (n = 13,722,784) in FASTA format from the GISAID repository (https://www.gisaid.org/) [8, 29, 30]. The dataset has been limited to sequences uploaded prior to the 3rd of November 2022 and included clades S, G, GH, GR, GRY, GV, GK, and GRA. Clades L and V were not present in the GISAID dataset, despite being documented in the literature. The spurious clade S with the count of 15,696 was considered an outlier to prevent significant data loss during subsequent rarefaction, given that clade S was not considered part of the evolutionary path of SARS-CoV-2 [31]. This decision aimed to prevent significant data loss during subsequent rarefaction and given that Clade S was not considered part of the evolutionary path of SARS-CoV-2. Additionally, clade O was omitted as it comprises the majority of unclassified sequences, introducing potential noise to the prediction model without providing any value to the evolutionary path.

To decrease the bias among bigger and smaller clades, resulting from the different number of sequences available in each clad, all clades were randomly stratified and subsampled equally according to the smallest clade GV using seqtk tool (https://github.com/lh3/seqtk). The 7 files (each representing one clade) were subsampled to 185,207 sequences per clade. Using a customized Python script, the continental origin was inferred from each strain FASTA header and incorporated in the metadata file using customized Python script. Genomes without a clear continental origin were labeled as unknown for subsequent analysis. For each genome, Di and Tri frequencies were calculated. For this purpose, we developed a C++ tool (GenoSig) capable of handling large collections of genomes in a computationally efficient manner, implementing the approach developed for the PaSiT tool in a manner that would parse our data without the need for additional steps. The produced frequencies incorporate all 16 Di and 64 Tri possible frequencies, leading to 80 frequencies signal which were named in our work as (Di and Tri). Of note, due to partial sequence or noise, any sequence file that did not produce a Di and Tri was further excluded from our analysis, leading to the final dataset (n = 1,131,185; Table 1a).

**Table 1:** Number of collected SARS-CoV-2 genomes in a) the main dataset (n = 1,131,185) b) the validation dataset (n = 67,399)

Clades	SARS-CoV-2 genomes	Continents	SARS-CoV-2 genomes
*(a) The main dataset (n* = *1,131,185)*			
Clade_G	163,511 (14.45%)	Africa	17,986 (1.59%)
Clade_GH	162,666 (14.38%)	Asia	87,711 (7.75%)
Clade_GK	154,275 (13.6%)	Europe	576,936 (51.00%)
Clade_GR	162,619 (14.37%)	North America	389,136 (34.4%)
Clade_GRA	159,190 (14.07%)	Oceania	10,761 (0.951%)
Clade_GRY	170,070 (15%)	South America	43,548 (3.84%)
Clade_GV	158,854 (14%)	Unknown	5107 (0.45%)
*(b) The validation dataset (n* = *67,399)*			
Clade_G	3161 (4.68%)	Africa	2225 (3.3%)
Clade_GH	6169 (9.15%)	Asia	12,145 (18%)
Clade_GK	22,436 (33.28%)	Europe	28,940 (42.93%)
Clade_GR	10,536 (15.63%)	North America	13,784 (20.35%)
Clade_GRA	17,844 (26.47%)	Oceania	1781 (2.64%)
Clade_GRY	6591 (9.77%)	South America	6761 (10.03%)
Clade_GV	662 (0.98%)	Unknown	1763 (2.61%)

To evaluate the robustness of our approach for clades and continental origins, we introduced an unexposed supplementary validation dataset, subsequently later than the main dataset, covering the submission period from the 4th of November 2022 to the 20th of November 2023. WGS were extracted in FASTA format from the GISAID repository using the "Search" module, selecting only the complete and high-coverage records. Metadata were extracted in the same fashion as the main dataset, leading to the validation dataset (n = 67,399; Table 1b). This dataset was then subjected to GenoSig to produce Di and Tri nucleotide frequencies in the same fashion as the main dataset.

### Machine learning, deep learning, and statistical analysis methods

Six supervised ML classifiers were used in this study, including Linear Support Vector Machine (SVM), Radial Kernel Support Vector Machine (RBF), Logistic Regression (LR), Naïve Bayes (NB), Decision Tree (DT), and Random Forest (RF). Additionally, a neural network architecture was used as a DL classifier model [31]. For the ML models, default parameters provided by scikit-learn were used across the board. For the RF model, parameters including 100 estimators, a random state of 42, and the "entropy" criterion [32]. For the DL model, it was implemented using the Keras library v(2.3.1) with a TensorFlow v(2.15.0) backend. The neural network included layers with 1024, 512, 256, 128, and 64 neurons, respectively. The final layer had a number of neurons equal to 7 which is the number of unique classes in the target variable, activated by the sigmoid function. The model was compiled using the Adam optimizer and employed the sparse categorical cross-entropy loss function.

It is crucial to highlight that the main dataset (n = 1,131,185) was split into an 80% training dataset and a 20% testing dataset for the ML and for the DL models (Additional file 1: Fig. S1). This partitioning strategy ensured that the models were trained on a substantial portion of the data, facilitating a more robust evaluation of their performance on the testing set. The training dataset underwent a tenfold cross-validation process. This approach involved dividing the training dataset into 10 subsets and iteratively training the model on 9 folds while validating with the remaining fold allowing shuffling in each iteration. This process was repeated 10 times, each time with a different validation fold.

In the main dataset, the performance report, including precision, recall, and F1-score, was generated using the scikit-learn library. For the validation dataset, a custom R v(4.3.2) script was employed. This script compared the ground truth value against the predicted values for each classifier. The evaluation metrics computed included overall accuracy, sensitivity, specificity, per-class balanced accuracy, and misclassifications between classes. The caret package v(6.0.94) was utilized for these calculations, and the results were utilized to generate a chord diagram through the circlize package v(0.4.15) and ggplot package v(3.4.4) [33–35]. PCA and Correlation coefficients were calculated using PCA() and corr() functions in pandas package.

As for the statistical analysis, a custom R script was utilized to conduct a normality test using the Shapiro–Wilk test. Subsequently, the *p-values* were adjusted with Bonferroni correction. To compare the mean averages of tenfold cross-validation of each group with the best model as a reference, a non-parametric comparison was performed using a pairwise Wilcoxon signed-rank test. Finally, the *P-value* was considered significant if (< 0.05). Regarding the used programming languages, apart from GenoSig, the rest of the work was written in Python (3.8) with dependencies from Pandas v(2.14) [36], Numpy v(1.26.0) [37], Scikit-Learn v(1.3.0) [32], and Matplotlib v(3.8.2) [38]. Finally, calculating the computational efficiency of the GenoSig tool and its comparison to other tools was done using a bash script on a PC with 24 GB RAM and Intel core i5-8265U CPU @ 1.60 GHz. A high-cluster computer was used for metadata generation and models’ training and prediction.

## Results

### The combined use of Di and Trinucleotide frequencies were able to train a robust model using random forest and deep learning approaches

**Fig. 1:**
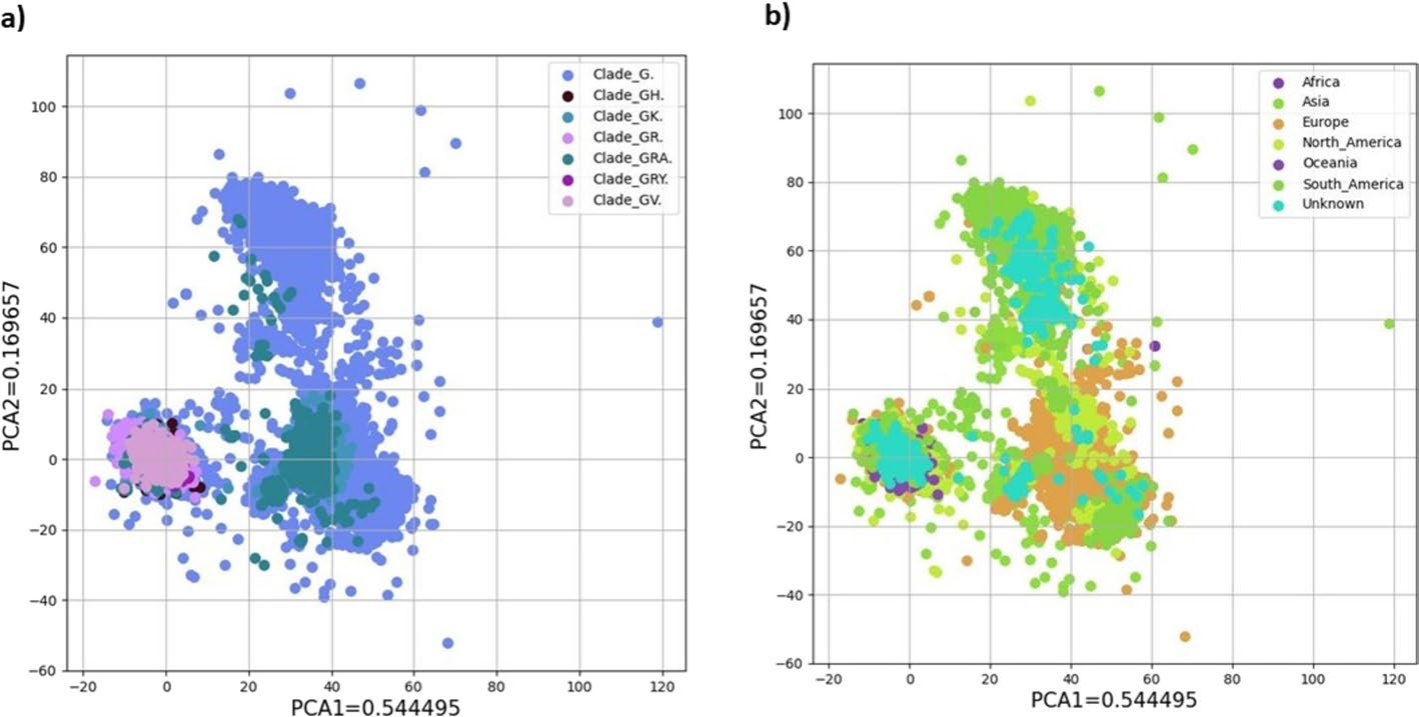
PCA exploratory analysis using GenoSig’s Di and Tri nucleotide frequencies matrix from the main data set for prediction of **a** clades and **b** continents

**Fig. 2:**
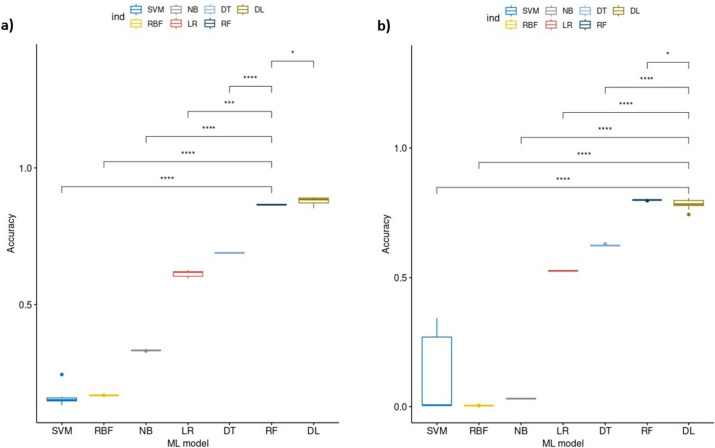
Comparing the -tenfold cross-validation accuracy of the ML/DL models on the main dataset for prediction of **a** clades and **b** continents

In our work, we wanted to utilize Di and Tri nucleotide frequencies to train our classifiers to be capable of identifying the clade origin. For this purpose, we first applied GenoSig to extract the Di and Tri nucleotide frequencies from the main dataset. Upon performing a principal component analysis (PCA) to evaluate the clustering potential of the dataset, it became apparent that our dataset displayed a non-linear nature. This observation is supported by the fact that 70% of the variance in the two principal components did not reveal clear clustering patterns with respect to clade or continent labels (Fig. 1). Then, we proceeded to compare the models in a systematic fashion, including Deep Learning (DL), Random Forest (RF), Decision Tree (DT), Logistic Regression (LR), Naïve Bayes (NB), Support vector with Radial Basis Function (RBF,) and Linear Support vector machine (SVM). The classifiers were trained and tested using a tenfold cross-validation fashion. From this analysis, DL achieved significantly the best performance with an accuracy of 87.88 (± 0.013), while the other ML models had a lower performance, with accuracy of 68.92% (± 0.001) for DT, 61.39% (± 0.01) for LR, 33.1% (± 0.0008) for NB, 16.4% (± 0.0003) for RBF and 15.7% (± 0.03) for SVM. Only RF had a performance close to DL with 86.37% (± 0.0009) (Table 2; Fig. 2a). For the remainder of the results in this section, we focused on the top-performing models, namely DL and RF, as they exhibit comparable performance yet possess distinct architectures.

**Table 2:** Comparing the tenfold cross-validation accuracy of the ML/DL models in the main dataset for clades and continents classification

Clades classification	tenfold cross-validation accuracy (± SD)	Continent classification	tenfold cross-validation accuracy (± SD)
SVM	15.7% (± 0.03)	SVM	11.1% (± 0.160)
RBF	16.4% (± 0.0003)	RBF	0.4% (± 0.0001)
NB	33.1% (± 0.0008)	NB	3.11% (± 0.0005)
LR	61.39% (± 0.01)	LR	52.6% (± 0.0006)
DT	68.92% (± 0.001)	DT	62.4% (± 0.002)
RF	86.37% (± 0.0009)	RF	79.92% (± 0.001)
DL	87.88 (± 0.013)	DL	78.34% (± 0.018)

For RF, it was shown that the best F1-score was for clade GRA 0.95, GK 0.92, GRY 0.91, GV 0.88, GH 0.81, GR 0.78,  and G 0.77. For precision, the same order as the F1-score, except that GRY 0.88 was lower than GV 0.9 and GR 0.81 was higher than GH 0.8. For recall, GRA 0.96 had the highest value and GR 0.74 had the lowest value. For DL, with the same order of best-performing clades like RF, we observed that the best F1-scores were for clade GRA 0.97, GK 0.95, GRY 0.93, GV 0.89, GH 0.84, GR 0.79 and G 0.79 respectively (Additional file 1: Table S1a). The precision mirrored the F1-score order with the exception that GH 0.89 surpassed GV 0.86, and GR 0.82 exceeded G 0.78. In terms of recall, GRA 0.97 achieved the highest value, while GR registered the lowest value at 0.77.

**Fig. 3:**
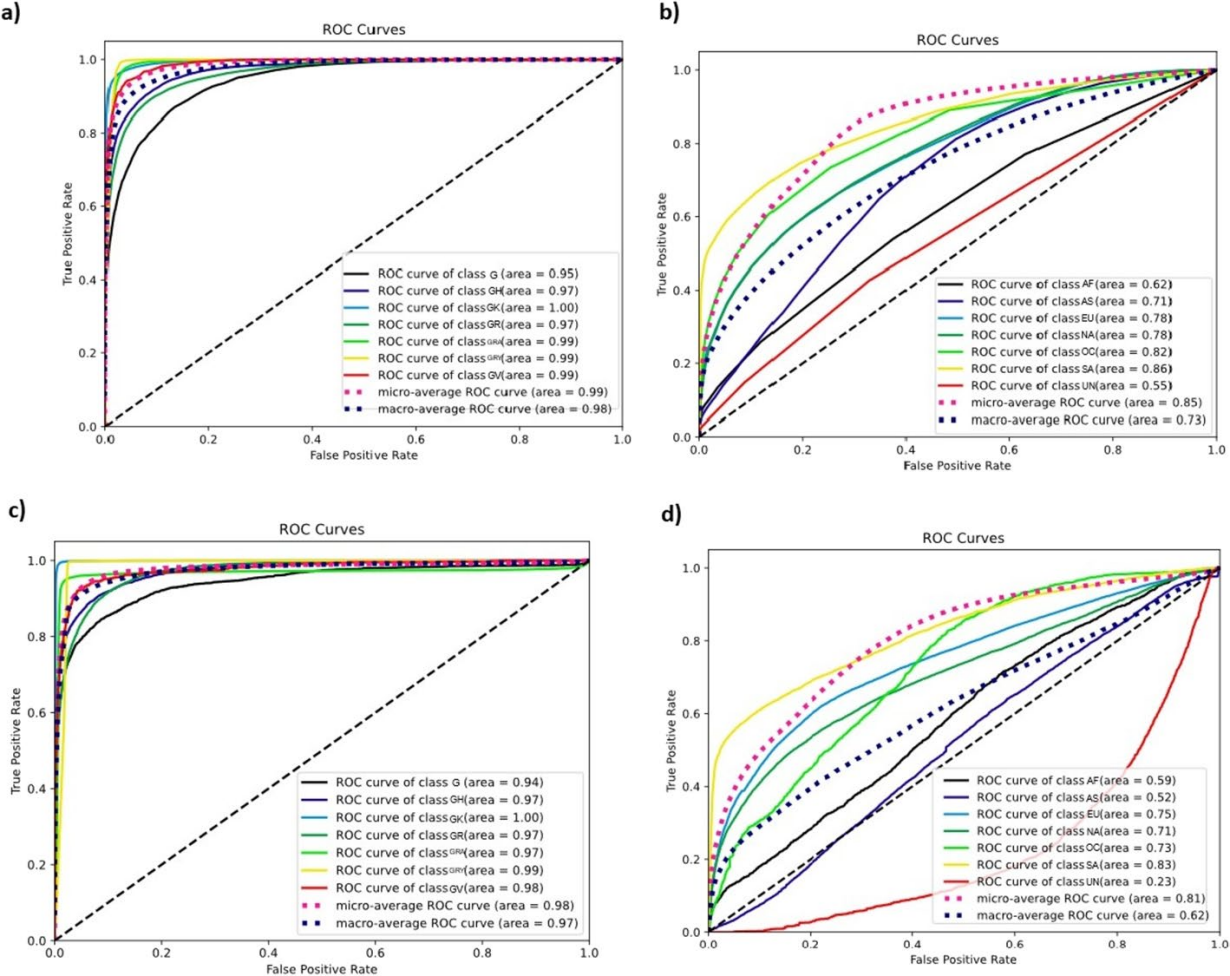
ROC-AUC curve based on the validation dataset using RF model on **a** clades and **b** continents also using DL model on **c** clades and **d** continents

**Fig. 4:**
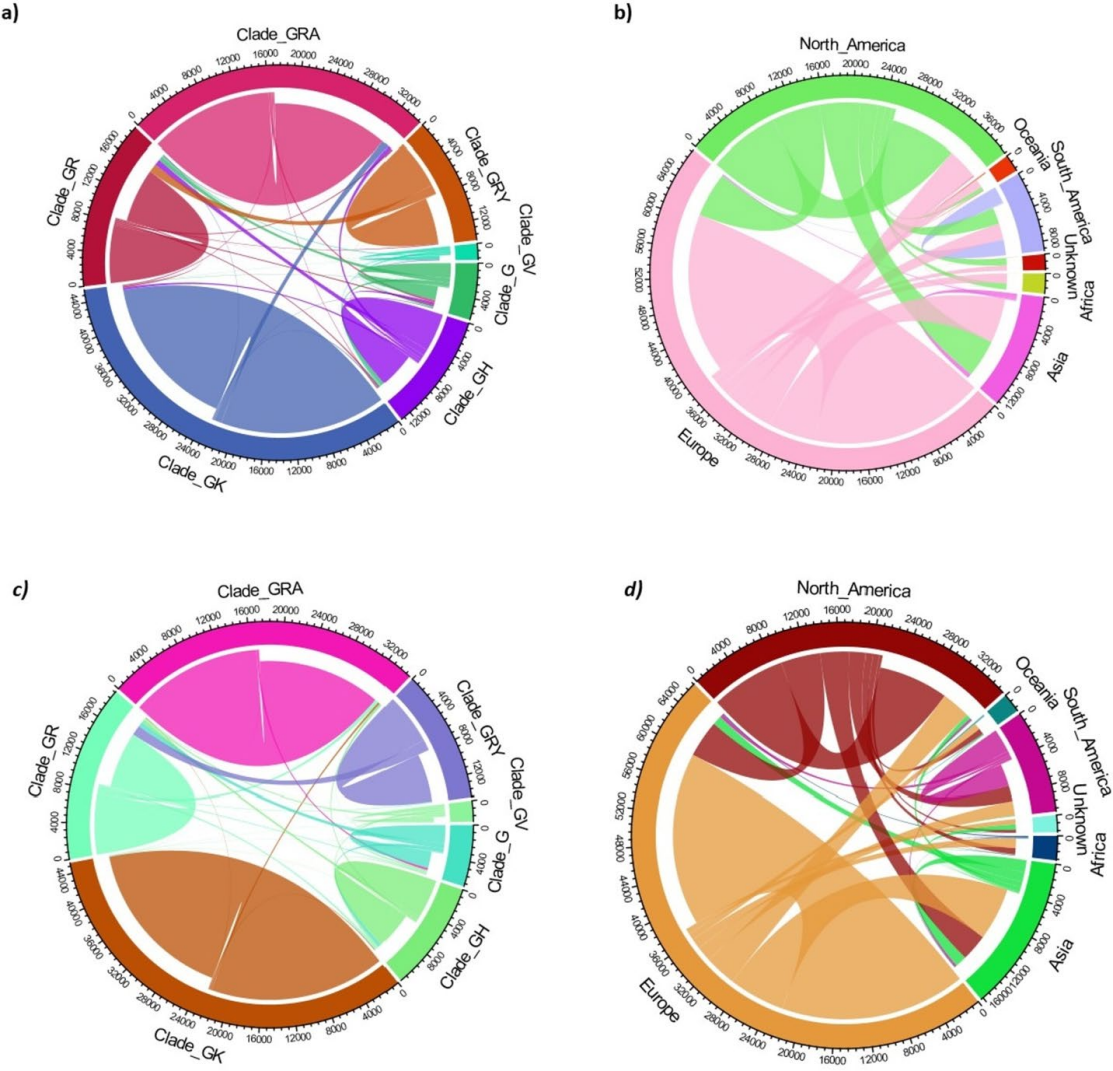
Chord diagram based on the confusion matrix of validation dataset and RF model **a** clades **b** continents and DL **c** clade **d** continents

To assess the robustness of our trained models using the validation dataset, we evaluated overall accuracy, sensitivity, specificity, per-class balanced accuracy, and ROC-AUC curves. The results indicated that DL achieved an overall accuracy of 90.4%, outperforming RF, which attained an overall accuracy of 87.76%. For both DL and RF, the clades GK, GRA, and GRY had the best-balanced accuracies, while clades G and GH had the worst-balanced accuracies. The detailed performance measures of both models and ROC-AUC from the validation dataset were reported and visualized (Table 3a; Fig. 3a, c). The misclassification biases were assessed in the confusion matrix for both RF and DL models based on the validation dataset. Regarding RF model misclassifications, 10.47% of clade G was misclassified as GH, and 8.11% of clade GH was misclassified as clade G. On the other side, 7.31% of clade G was the highest misclassified as clade GR, and 5.79% of clade GR was misclassified as clade G. Also, 11.33% of clade GV had the highest misclassification as clade GH, and 11.23% of clade GR was misclassified as clade GRY. The detailed misclassifications are reported and visualized in (Table 4a; Fig. 4a). Regarding DL model misclassification, 7.28% of clade GH was misclassified as G, and 3.42% of clade G was misclassified as clade GH. On the other side, 6.67% of clade GR was misclassified as clade G and 5.82% of clade G was misclassified as clade GR. Also, 12.97% of clade GR was the highest misclassification as clade GRY. The detailed misclassifications are reported and visualized in (Table 5a; Fig. 4c).

**Table 3:** Performance report of RF and DL models among validation dataset for prediction of a) clades b) continents

	RF			DL		
	Sensitivity	Specificity	Balanced Accuracy	Sensitivity	Specificity	Balanced Accuracy
*(a) Model/clades*						
Clade_G	0.614	0.98	0.797	0.713	0.979	0.846
Clade_GH	0.802	0.965	0.884	0.809	0.986	0.897
Clade_GK	0.966	0.971	0.968	0.991	0.989	0.99
Clade_GR	0.716	0.983	0.849	0.727	0.979	0.853
Clade_GRA	0.901	0.984	0.943	0.936	0.992	0.964
Clade_GRY	0.973	0.979	0.976	0.979	0.977	0.978
Clade_GV	0.803	0.99	0.897	0.909	0.985	0.947
*(b) Model/continents*						
Africa	0.006	0.999	0.503	0.025	0.996	0.51
Asia	0.052	0.994	0.523	0.12	0.952	0.536
Europe	0.774	0.581	0.678	0.771	0.605	0.688
North America	0.672	0.698	0.685	0.655	0.771	0.713
Oceania	0.042	0.998	0.52	0.058	0.998	0.528
South America	0.316	0.997	0.656	0.424	0.981	0.703

**Table 4:** Confusion matrix of RF model on validation dataset for prediction of a) clades and b) continents

Prediction/Ref	Clade_G (%)	Clade_GH (%)	Clade_GK (%)	Clade_GR (%)	Clade_GRA (%)	Clade_GRY (%)	Clade_GV (%)
*(a)*							
Clade_G	61.47	8.11	0.29	5.79	0.21	0.46	4.53
Clade_GH	10.47	80.27	1.40	7.91	3.00	0.14	11.33
Clade_GK	2.97	2.33	96.60	0.85	5.42	0.03	0.91
Clade_GR	7.31	3.39	0.79	71.63	1.15	1.67	2.42
Clade_GRA	10.72	1.82	0.76	1.03	90.13	0.36	0.00
Clade_GRY	1.04	0.44	0.02	11.23	0.06	97.34	0.45
Clade_GV	6.01	3.65	0.15	1.56	0.02	0.00	80.36

Prediction/Ref	Africa (%)	Asia (%)	Europe (%)	North_America (%)	Oceania (%)	South_America (%)	Unknown (%)
*(b)*							
Africa	0.67	0.01	0.01	0.00	0.00	0.00	0.00
Asia	0.45	5.29	0.52	0.46	1.52	0.25	1.19
Europe	61.80	51.12	77.48	31.33	65.24	33.90	41.52
North America	36.94	43.42	21.65	67.25	28.69	34.00	56.55
Oceania	0.00	0.01	0.00	0.56	4.21	0.21	0.00
South America	0.13	0.16	0.34	0.39	0.00	31.64	0.34
Unknown	0.00	0.00	0.00	0.00	0.34	0.00	0.40

**Table 5:** Confusion matrix of DL model on the validation dataset for prediction of **a** clades and **b** continents

Prediction/Ref	Clade_G (%)	Clade_GH (%)	Clade_GK (%)	Clade_GR (%)	Clade_GRA (%)	Clade_GRY (%)	Clade_GV (%)
*(a)*							
Clade_G	71.34	7.28	0.12	6.67	0.45	0.36	3.78
Clade_GH	3.42	80.92	0.22	3.82	1.40	0.03	2.72
Clade_GK	0.44	1.70	99.18	0.63	1.54	0.00	0.76
Clade_GR	5.82	4.49	0.25	72.70	2.82	1.65	1.81
Clade_GRA	9.74	0.02	0.04	0.34	93.66	0.00	0.00
Clade_GRY	0.35	0.13	0.00	12.97	0.00	97.92	0.00
Clade_GV	8.89	5.46	0.17	2.88	0.13	0.03	90.94

Prediction/Ref	Africa (%)	Asia (%)	Europe (%)	North_America (%)	Oceania (%)	South_America (%)	Unknown (%)
*(b)*							
Africa	2.52	0.63	0.49	0.13	0.22	0.07	0.40
Asia	3.06	12.07	4.07	4.13	5.33	0.99	37.10
Europe	56.54	56.24	77.19	27.54	45.03	27.05	38.06
North America	36.49	28.70	16.58	65.59	43.51	29.18	21.89
Oceania	0.04	0.07	0.03	0.54	5.90	0.24	0.06
South America	1.35	2.29	1.63	2.07	0.00	42.43	2.50
Unknown	0.00	0.00	0.00	0.00	0.00	0.03	0.00

Analyzing the feature importance of our trained classifiers revealed distinct approaches to feature selection by RF and DL. Both models incorporated all 80 Di and Tri nucleotide features but assigned varying degrees of importance to each. Notably, the RF model identified (CGG, CCG, GGG, CCC, CTG) as the top five crucial features, (Additional file 1: Fig. S2a). Conversely, the DL model prioritized (CG, CC, GG, GGA, TCG) as the top three significant features (Additional file 1: Fig. S2c). To confirm these findings, we retrained the two models on the validation dataset using Di only, Tri only, or both Di and Tri as inputs for training. In this analysis, the RF classifier significantly favored the combined signal over Tri only and over Di alone. However, for DL, although it preferred the combined signal like RF, it demonstrated a significant preference for the Di signal over the Tri signal (Additional file 1: Fig. S3a, c).

### Reduced efficacy of random forest and deep learning models in tracing continental origin through Di and Tri nucleotide frequency analysis in our dataset

In the second part of our work, RF demonstrated a significant performance advantage over other classifiers for the prediction of continental origin, including DL, in the tenfold cross-validation accuracy. In detail, RF achieved an accuracy of 79.92% (± 0.001), while DL achieved a slightly lower accuracy of 78.34% (± 0.018). As anticipated, the performance of the other ML models was notably lower, with DT with 62.4% (± 0.002), LR with 52.6% (± 0.0006), SVM with 11.1% (± 0.160), NB with 3.11% (± 0.0005), and RBF with 0.4% (± 0.0001) (Table 2; Fig. 2b). As with the clades analysis, we investigated underlying performance of both comparable models. For the RF classifier, F1-score for the prediction of continental origin was the highest in Europe with 0.85. While with North America it was 0.77 and 0.64 for South America, 0.63 for Asia, 0.61 for Oceania, and 0.28 for Africa. For precision, surprisingly, Oceania comes first with 0.96, Asia 0.89, Africa 0.89, South America 0.81, Europe 0.80 and finally North America 0.76. For recall, a similar order to F1-score as Europe comes first with 0.90, and the lowest value was Africa with 0.17. The results of the DL classifier were similar but not identical to that of the RF classifier. The F1-score for predicting continental origin was the highest in Europe, registering at 0.82. North America followed with a score of 0.78, South America at 0.66, Oceania at 0.66, Asia at 0.59, and Africa at 0.18. As for the precision, Oceania came first with 0.92, followed by Europe (0.82), North America (0.76), Asia (0.74), South America (0.70), and finally Africa with a precision of 0.14. For recall, a similar order to the F1-score with Europe having the highest performance (0.83) and Africa having the least performance (0.27; Additional file 1: Table S1b).

As with the clade analysis, we evaluated the robustness using the validation dataset. Both models experienced an overall drop in accuracy, with RF achieving an overall 51.29%, while DL exhibited a slightly higher overall accuracy of 53.23%. Inside each continent, like the main dataset, and for both models, Europe, North America, and South America had the best-balanced accuracies over other continents. The detailed performance measures of both models and ROC-AUC in the validation dataset for the prediction of continental origin are reported and visualized in (Table 3b; Fig. 3b, d).

With respect to misclassification in the validation dataset, it was clear that all continents were most commonly misclassified as Europe by the RF model, with the exception of South America where 34% was highly misclassified as North America. The highest misclassified continent was Africa as 61.80% of Africa’s records were misclassified as Europe, while 36.94% were misclassified as North America. The detailed misclassifications are reported and visualized in (Table 4b; Fig. 4b). For DL model misclassification, the same pattern as RF was observed. All continents were highly misclassified as Europe with same exception of South America where 29% of its signals were misclassified as North America. Similarly, the highest misclassified continent was Africa as 56.54% of Africa was misclassified as Europe, while 36.49% of Africa was misclassified as North America. The detailed misclassifications are reported and visualized in (Table 5b; Fig. 4d).

With respect to the feature selection per continent, distinctions in the feature importance of RF and DL models were also observed. Again, both models encompassed all 80 Di and Tri nucleotide features. Notably, the RF model identified (CG, TCC, GC, CCT, GGA) in the top five features (Additional file 1: Fig. S2b), whereas the DL model prioritized (CG, GG, CC, GA, TC) (Additional file 1: Fig. S2d). Re-training the model with the validation dataset, using each signal (Di or Tri) separately or combined, revealed a consistent pattern for both RF and DL. In both models, there was a significant preference for the combined signal over the separate signals of Di or Tri. Additionally, the observed pattern indicated that RF tends to significantly favor the Tri signal over Di, while DL exhibits the opposite preference, favoring the Di signal over Tri (Additional file 1: Fig. S3b, d).

### Software implementation

**Fig. 5:**
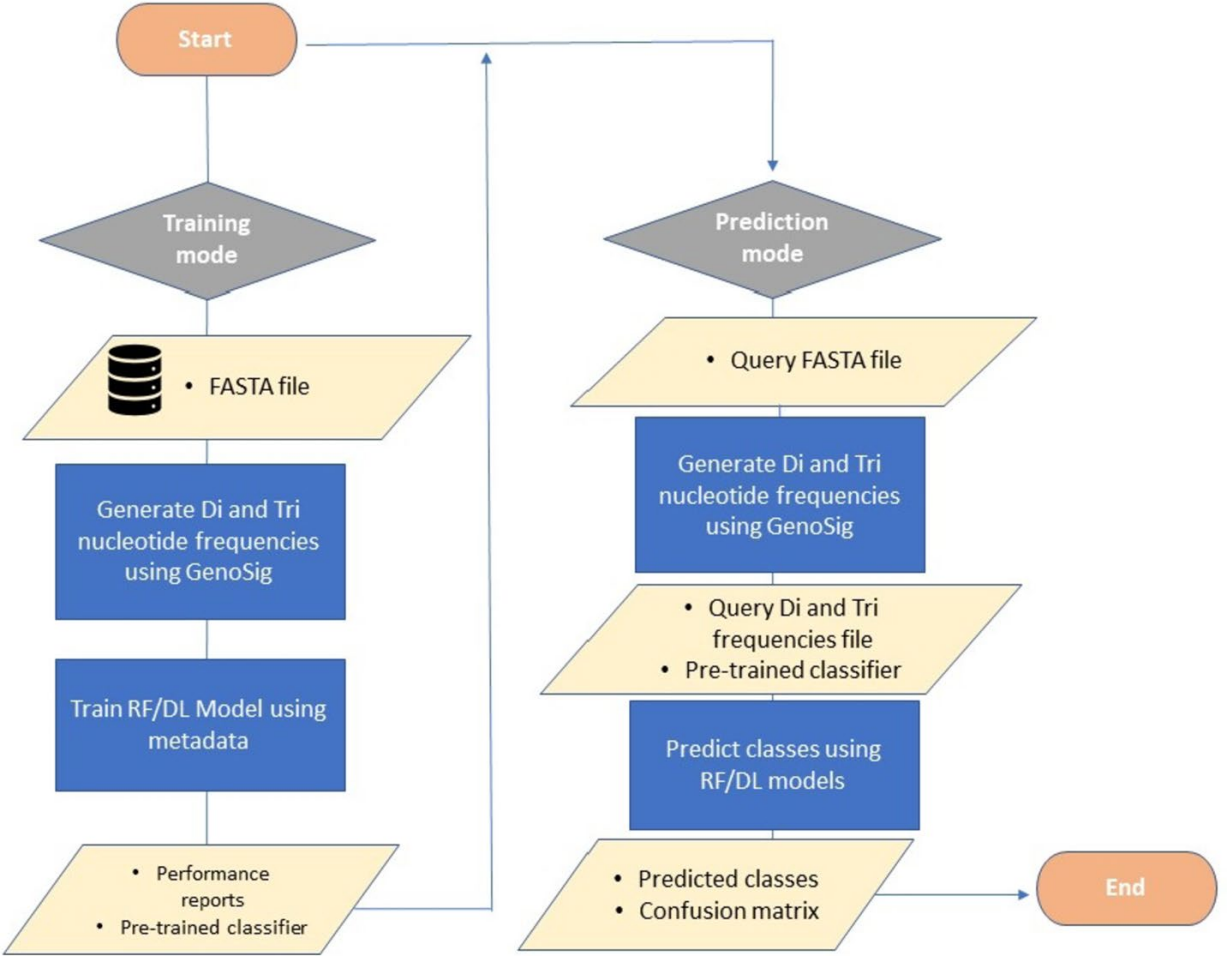
The schematic workflow of our approach employing GenoSig alongside the ML or DL models

Various tools are available for extracting k-mer nucleotide frequencies from FASTA or FASTQ files (Additional file 1: Table S2) [39–41]. To our knowledge, only SeekR library can generate a frequency signal per header/contig, aligning with our feature extraction needs for classification tasks. However, SeekR was written in an interpreted language (Python 3.8). Therefore, PasiT was modified to incorporate this functionality with reduced additional time and fewer options, resulting in the release of GenoSig as a tool to produce huge files or datasets. Highlighting the superior performance of C++ over Python, GenoSig demonstrated improved RAM and CPU efficiency compared to SeekR when handling three distinct FASTA files (5.4 GB, 1.1 GB, and 245.4 MB). For the 5.4 GB FASTA file, GenoSig required 4.1 min, utilized 9.3 GB of RAM, and used 40% of the CPU. In contrast, SeekR took 47.8 min, utilized 16.1 GB of RAM, and consumed 88% of the CPU across all 8 available processors (Additional file 1: Fig. S4). For the main dataset ~ 37.8 GB, GenoSig took 7 min and 33.364 s when measured as wall-clock time, 5 min and 59.671 s in terms of user time, and 1 min and 18.425 s as system time. In our study, the usage of GenoSig involves employing a small Perl script executed in a BASH terminal that requires no installation. This script calculates 80 Di and Tri nucleotide frequencies per genome. The resulting frequency matrix can be passed to the RF or DL pipeline, as shown in our workflow where GenoSig was used to generate signals for training a classifier in the training mode. Alternatively, it can produce a signal from a query sequence, which can be supplemented to a pre-trained classifier for predicting its class (e.g. origin) (Fig. 5).

### Discussion

Amid the dramatic spread of SARS-CoV-2, many projects started tracking the evolution of this pandemic, a process known for being computationally expensive, time-consuming,  and requiring dedicated algorithms for clustering or phylogenetics. The main bottleneck in this process is the sequence alignment followed by standard phylogenetic analysis [42]. This challenge was not exclusive to SARS-CoV-2 alone; it also encompassed other kinds of outbreaks. Hence, Several studies hypothesized that an alignment-free approach might be more effective to investigate the variations within large-scale genomic data, providing insights into the dynamics of evolution [43, 44]. Dlamini et al*.* showed that Dinucleotide frequencies can be used for classifying 7 viral species including SARS-CoV-2 and exploited the potential of this approach to classify SARS-CoV-2 according to their continental origin [20]. However, their work was done on a small scale of data (n = 32,899 sequences), and they did not provide a standard open-source tool to handle similar research questions. In our research, we extended their findings by scrutinizing the phylogenetic utility of 80 nucleotide frequencies, including 16 Di nucleotide frequencies (e.g., AA, AT, AC, etc.) and 64 Tri nucleotide frequencies (e.g., AAA, AAT, ATA, etc.). These were considered as signatures for an alignment-free approach to sequence comparison, and we assessed their classification potential using various ML or DL models. This was implemented in our newly introduced tool named GenoSig, which was assessed using a large collection of SARS-CoV-2 sequences (n = 1,131,185) in order to identify the clade and continental origin.

In our study, we acquired genome sequences submitted before November 3, 2022, employing rarefaction subsampling to balance the main dataset based on clades. We highlighted the superior performance of our DL and random RF implementations using Di and Tri nucleotide frequencies incorporated through GenoSig, compared to other classifiers. Particularly in clade classification, DL significantly outperformed RF. The performance analysis of clades involving DL and RF showcased the capability of the two classifiers to distinguish later clades, such as GRA, GK, and GRY, over earlier ones like GH, GR, and G. To validate the robustness of our clade prediction approach, we challenged our RF and DL models with the data submitted subsequent to our main dataset. From the validation dataset, our models showed a comparable performance to the main dataset in RF and DL models, in the validation dataset, G and GH showed predominant confusion. Furthermore, the highest misclassification was for clade GR to GRY in both models. Importantly, this misclassification aligns with the phylogeny of the two clades [45, 46]. The superior performance observed in later clades may be attributed to the cumulative accumulation of mutations over time, including both synonymous and non-synonymous mutations. This accumulation could be influenced by factors like host adaptation or, evasion strategies, such as those reported in the case of the GRA clade related to vaccination [11].

In the continental analysis, RF outperformed DL in the tenfold-cross-validation accuracy, yet both models demonstrated higher accuracy for Europe, North America, and South America compared to other continents. In the validation dataset, despite lower overall accuracy, DL had an edge over RF. In the confusion matrix, both models exhibited a similar bias towards Europe, North America, and South America, struggling to detect the less represented continents, particularly Africa. Interestingly, in the validation dataset misclassifications, both models tended to misclassify most continents as Europe, except for South America, which was misclassified as North America. This pattern may align with the geographic distance and travel dynamics between these two continents or could reflect an inherent bias in the validation dataset.

These findings suggest that the classifiers trained on the Di and Tri nucleotide frequencies for continent prediction did not achieve the same level of performance as the clade classifiers. This could be attributed to the fact that clades are already phylogenetically predefined and balanced. On the other hand, the continent data suffered from technical imbalances within datasets as well as the influence of epidemiological factors such as increased travel rates within and outside Europe and North America, especially with the border reopening. As a result, optimizing continental analysis is complex due to high sequencing capacity and high incidents in Europe and North America, resulting in higher numbers of available sequences [47]. Furthermore, it requires a careful method considering, the evolving time dynamics of SARS-CoV-2 clades to eliminate time as a confounder. In our analysis of feature importance for clade and continent classification, both DL and RF utilized all 80 Di and Tri nucleotide features with diverse weights, though the pattern of correlation in nucleotide frequencies (Additional file 1: Fig. S5). The top features were prominently associated with cytosine and guanine, confirming the significance of cytosine in the evolution of SARS-CoV-2 [48]. Additionally, there are reports indicating that mutational asymmetries affect the hydrophobicity of the virus proteins for clades over time [49]. This alignment underscores the relevance of Di and Tri nucleotide frequencies in the classification process, linking genetic variations with evolutionary dynamics or maybe functional characteristics of the virus.

## Conclusion

Our work introduced a reductive yet fast and robust approach for predicting SARS-CoV-2 clades, utilizing Di and Tri nucleotide frequencies and employing either DL or RF as the model of choice. Both DL and RF achieved quite comparable results, albeit relying on very distinct architectures. Additionally, we emphasize the significance of considering misclassifications as an indicator of model logic and for epidemiological interpretation. This approach was implemented using our tool entitled GenoSig. Given the recent expansion of genomic datasets, such an approach can be extended to address various epidemiological questions related to viral or bacterial genomes, as well as meta-genome analysis. GenoSig’s smooth performance enables adaptability to various hardware capabilities. As a future direction, it would be interesting to expand the approach to investigate more species employing Di and Tri nucleotide signals.

### Supplementary Information

**Additional file 1:**.** Table S1.** RF model and DL performance measures of different** a** clades** b** continents in the main dataset.** Table S2.** Comparison of K-mer frequencies tools similar to GenoSig.** Fig. S1.** Summary graph showing used datasets splitting approach for training and testing, allowing shuffling in each iteration.** Fig. S2.** Features importance for RF** a** clades** b** continents. for DL** c** clades** d** continents. From the models that were trained on the main dataset.** Fig. S3.** Employing the validation dataset, re-training model using Di nucleotide only, Tri nucleotide only and combined signal Di and Tri on RF model** a** clades** b** continents and DL** c** clades** d** continents.** Fig. S4.** Comparing GenoSig versus SeekR in terms of** a** Time (minutes),** b** CPU%, **c** Memory (GB).** Fig. S5.** Summary graph showing correlation (0,1) among the 80 Di and Tri frequency produced from the main dataset.

## Data Availability

The GenoSig script, machine and deep learning models, data analysis scripts, and examples of the data used in this project can be accessed via (https://github.com/AhmedElsherbini/Code_for_Elsherbini_et_al_2023).
