# Supplementary material for: Utilizing genomic signatures to gain insights into the dynamics of SARS-CoV-2 through Machine and Deep Learning techniques
Source: BMC Bioinformatics. 2024 Mar 27;25:131. doi: 10.1186/s12859-024-05648-2 (PMC10967124; doi:10.1186/s12859-024-05648-2)
Supplement: Supplementary file 1 — Additional file 1:. Table S1. RF model and DL performance measures of different a clades b continents in the main dataset. Table S2. Comparison of K-mer frequencies tools similar to GenoSig. Fig. S1. Summary graph showing used datasets splitting approach for training and testing, allowing shuffling in each iteration. Fig. S2. Features importance for RF a clades b continents. for DL c clades d continents. From the models that were trained on the main dataset. Fig. S3. Employing the validation dataset, re-training model using Di nucleotide only, Tri nucleotide only and combined signal Di and Tri on RF model a clades b continents and DL c clades d continents. Fig. S4. Comparing GenoSig versus SeekR in terms of a Time (minutes), b CPU%, c Memory (GB). Fig. S5. Summary graph showing correlation (0,1) among the 80 Di and Tri frequency produced from the main dataset. [file 12859_2024_5648_MOESM1_ESM.docx]

# **Utilizing genomic signatures to gain insights into the dynamics of SARS-CoV-2 through machine and deep learning techniques.**

Ahmed M. A. Elsherbini ^1^, Amr Hassan Elkholy ^1^, Youssef M. Fadel ^1^, Gleb Goussarov ^2^, Ahmed Mohamed Elshal ^1^, Mohamed El-Hadidi^1^, Mohamed Mysara ^1,2*^

^1^ Bioinformatics group, Center for Informatics Science, School of Information Technology and Computer Science, Nile University, Giza, Egypt

^2^ Microbiology Unit, Belgian Nuclear Research Centre (SCK•CEN), Mol, Belgium

***Corresponding email:** [mmaysara@nu.edu.eg](mailto:mmaysara@nu.edu.eg)

**Supplementary tables**

**Supplementary Table 1. RF model and DL performance measures of different a) clades b) continents in the main dataset**

**a)**

| **Model / Clades** | **RF** | | | **DL** | | |
| --- | --- | --- | --- | --- | --- | --- |
|  | **precision** | **recall** | **F1-score** | **precision** | **recall** | **F1-score** |
| **Clade_G** | **0.77** | **0.76** | **0.77** | **0.78** | **0.8** | **0.79** |
| **Clade_GH** | **0.8** | **0.81** | **0.81** | **0.89** | **0.8** | **0.84** |
| **Clade_GK** | **0.91** | **0.93** | **0.92** | **0.94** | **0.95** | **0.95** |
| **Clade_GR** | **0.81** | **0.74** | **0.78** | **0.82** | **0.77** | **0.79** |
| **Clade_GRA** | **0.94** | **0.96** | **0.95** | **0.98** | **0.97** | **0.97** |
| **Clade_GRY** | **0.88** | **0.94** | **0.91** | **0.9** | **0.95** | **0.93** |
| **Clade_GV** | **0.9** | **0.87** | **0.88** | **0.86** | **0.92** | **0.89** |

**b)**

| **Model /  Continents** | **RF** | | | **DL** | | |
| --- | --- | --- | --- | --- | --- | --- |
|  | **precision** | **recall** | **F1-score** | **precision** | **recall** | **F1-score** |
| **Africa** | **0.89** | **0.17** | **0.28** | **0.14** | **0.27** | **0.18** |
| **Asia** | **0.89** | **0.49** | **0.63** | **0.74** | **0.49** | **0.59** |
| **Europe** | **0.8** | **0.9** | **0.85** | **0.82** | **0.83** | **0.82** |
| **North America** | **0.76** | **0.78** | **0.77** | **0.76** | **0.8** | **0.78** |
| **Oceania** | **0.96** | **0.45** | **0.61** | **0.92** | **0.51** | **0.66** |
| **South America** | **0.81** | **0.52** | **0.64** | **0.7** | **0.63** | **0.66** |
| **Unknown** | **0.86** | **0.12** | **0.21** | **0.64** | **0.08** | **0.15** |

**Supplementary Table 2. Comparison of K-mer frequencies tools similar to GenoSig.**

|  | GenoSig | SeekR | PaSiT/GenDiscal | Seqrequester | Jellyfish | MerCat2 |
| --- | --- | --- | --- | --- | --- | --- |
|  | Our work GitHub repo | https://github.com/CalabreseLab/seekr | https://github.com/LM-UGent/GenDisCal | https://github.com/marbl/seqrequester | https://github.com/gmarcais/Jellyfish | <https://github.com/raw-lab/mercat2> |
| Language | C++ | Python | C++ | C | C++ | Python |
| Aim of the tool | Di- and/or Trinucleotide (k-mer) frequencies. | Classification of nucleotide sequences based on k-mer frequencies. | Bacterial genome distance Calculator based on modified (k-mer) signature. | 1- Histogram of sequence lengths.  2- GC3. di- and trinucleotide kmer. | K-mer counter | 1- K-mer counter.  2- K-mer diversity estimator. |
| Input | FASTA | FASTA | FASTA | FASTA/ FASTQ | FASTA/ FASTQ | FASTA/ FASTQ |
| Output | Signal per contig | Signal per contig | Signal per file | Signal per file | Signal per file | Signal per file |
| Reference |  | (39) | (28) | N/A | (40) | (41) |

**Supplementary figures**

**
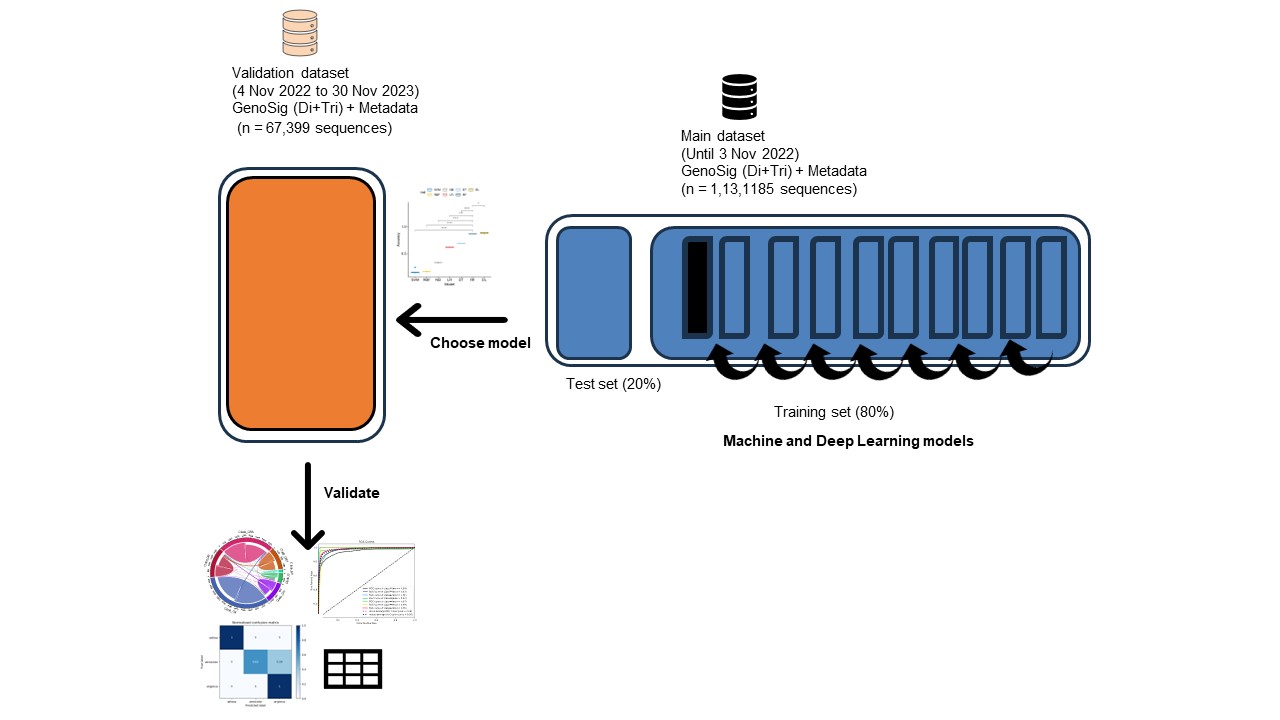
**

**Supplementary Fig. 1 Summary graph showing used datasets splitting approach for training and testing, allowing shuffling in each iteration.**

***
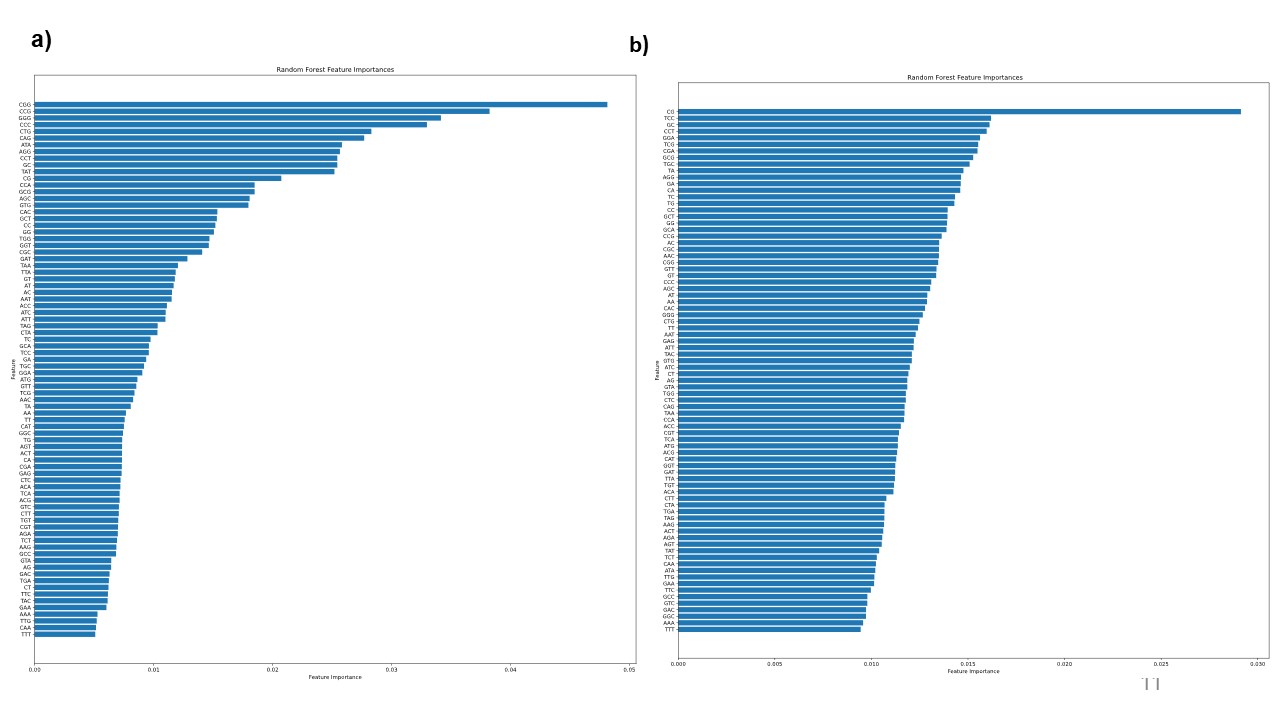
***

**Supplementary Fig. 2
Features importance for RF a) clades b) continents. for DL c) clades d) continents. From the models that were trained on the main dataset.**


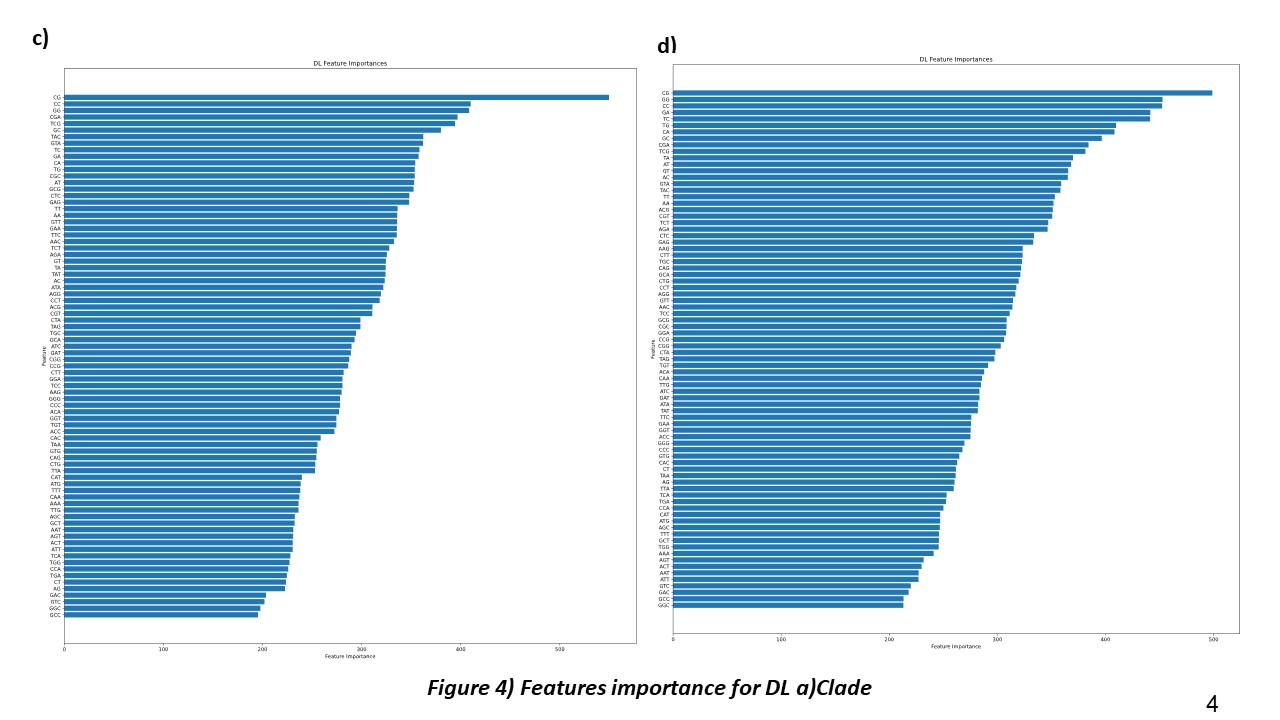


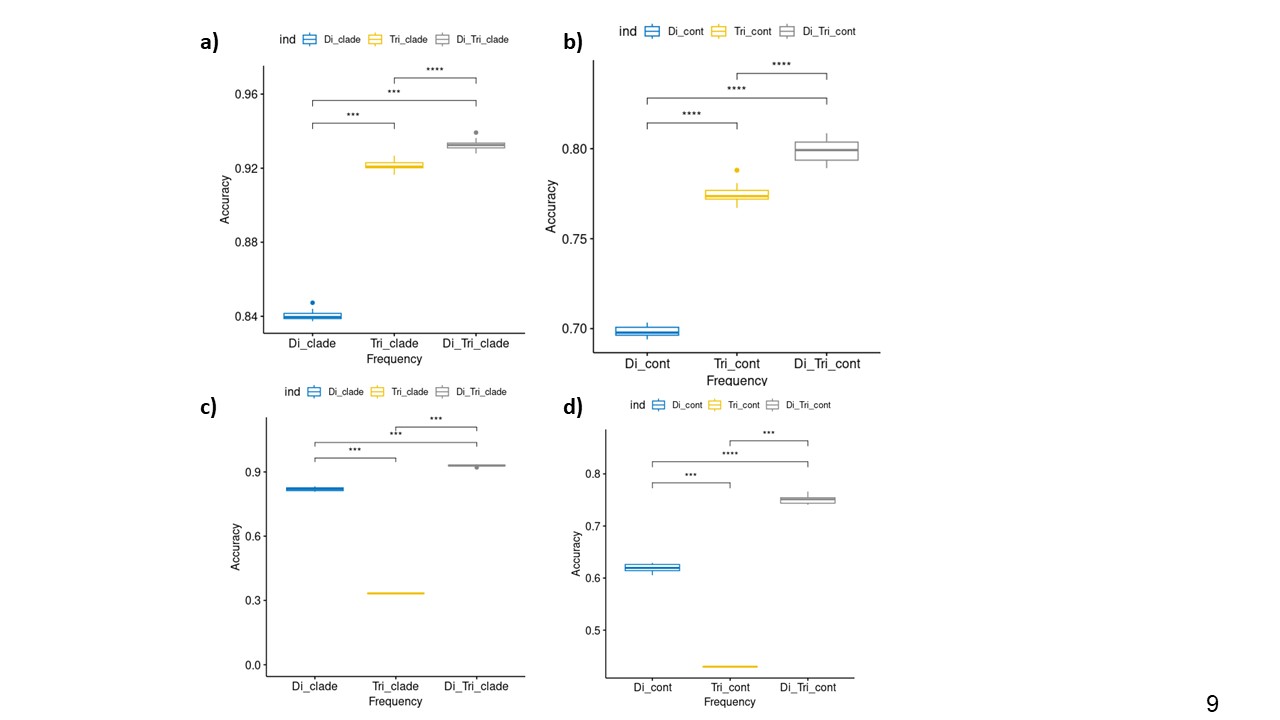


**Supplementary Fig. 3 Employing the validation dataset, re-training model using di nucleotide only, tri nucleotide only and combined signal Di and Tri on RF model a) clades b) continents and DL c) clades d) continents.**

***
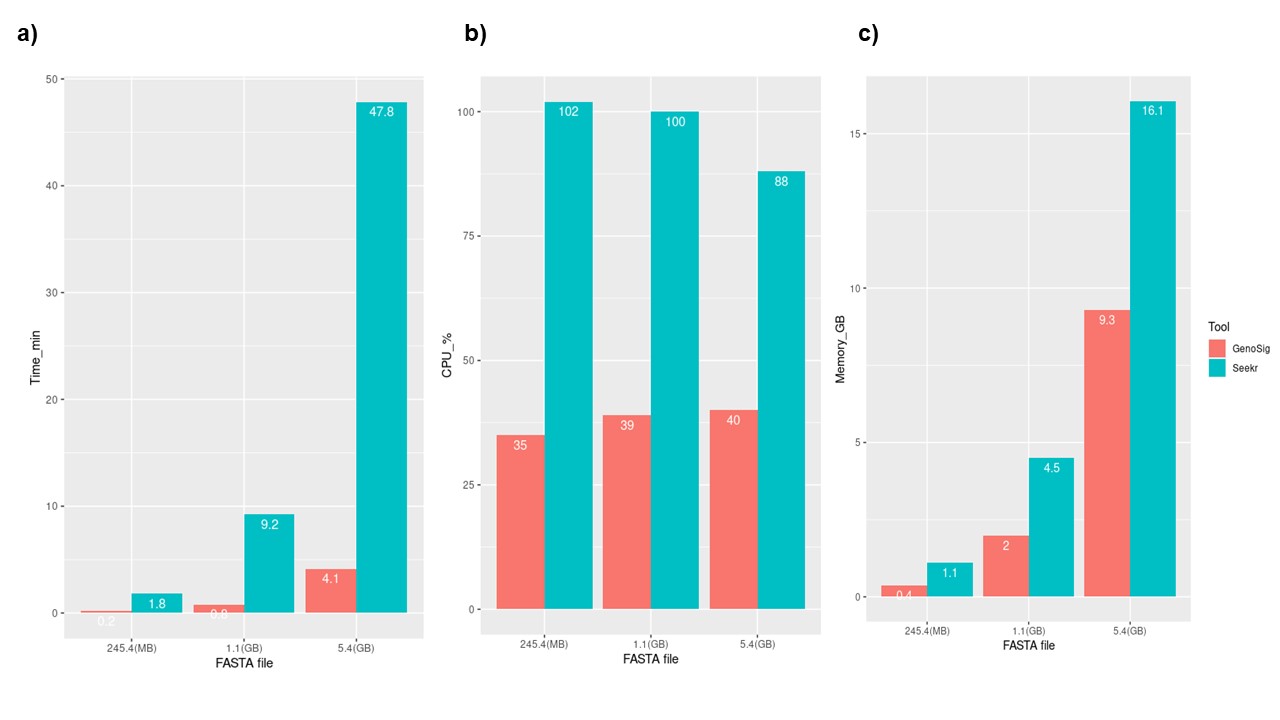
***

**Supplementary Fig. 4 Comparing GenoSig versus SeekR in terms of a) Time (minutes), b) CPU%, C) Memory (GB)**


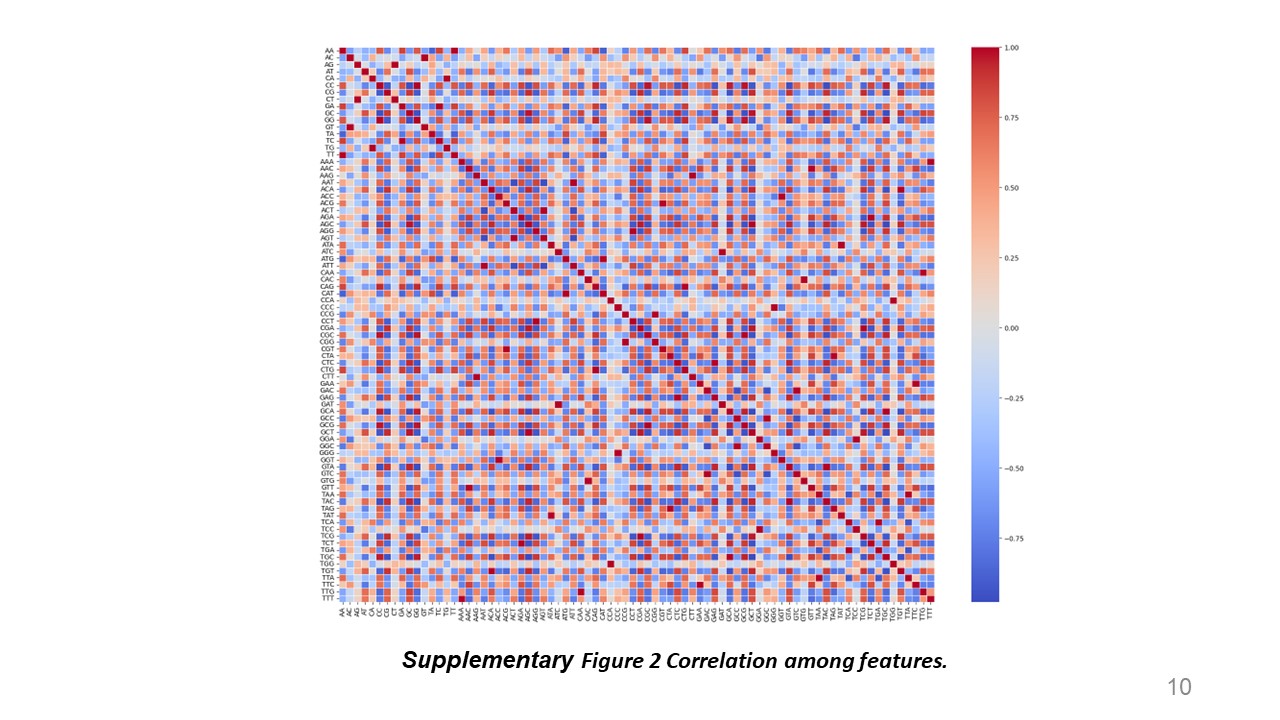


**Supplementary Fig 5. Summary graph showing correlation (0,1) among the 80 Di and Tri frequency produced from the main dataset.**
